# Accurate quantification of homologous recombination in zebrafish: brca2 deficiency as a paradigm

**DOI:** 10.1038/s41598-017-16725-3

**Published:** 2017-11-28

**Authors:** Jeroen Vierstraete, Andy Willaert, Petra Vermassen, Paul J. Coucke, Anne Vral, Kathleen B. M. Claes

**Affiliations:** 1 Center for Medical Genetics Ghent, Ghent University Hospital, Ghent, Belgium; 2 Department for Basic Medical Sciences, Ghent University, Ghent, Belgium

## Abstract

Homologous Recombination (HR) repair is essential for repairing DNA double strand breaks (DSB) in dividing cells and preventing tumorigenesis. BRCA2 plays an important role in HR by recruiting the DNA recombinase RAD51 to the DSB. Despite being a popular model organism in genetic and cancer research, knowledge on the conservation of the HR pathway and function of zebrafish Brca2 is limited. To evaluate this, we developed a Rad51 foci assay in zebrafish embryos. We identified the zebrafish embryonic intestinal tissue as an ideal target for Rad51 immunostaining. After inducing DSB through irradiation, Rad51 foci were present in irradiated embryos but not in unirradiated controls. We present a method for accurate quantification of HR. Both morpholino-induced knockdown and knockout of Brca2 lead to almost complete absence of Rad51 foci in irradiated embryos. These findings indicate conserved function of Brca2 in zebrafish. Interestingly, a statistically significant decrease in Rad51 foci was observed in Brca2 heterozygous carriers compared to wild types, indicative of haploinsufficiency, a hypothesised cause of some tumours in patients with a germline *BRCA2* mutation. In conclusion, we demonstrated the suitability of zebrafish as an excellent *in vivo* model system for studying the HR pathway and its functionality.

## Introduction

In every cell, the genomic material is constantly being damaged by exogenous or endogenous mechanisms^1^. Double Strand Breaks (DSB) are especially pathogenic, as there is no complementary base template available for accurate repair. These DSB can be repaired through two pathways: Non-Homologous End Joining (NHEJ) and Homologous Recombination (HR)^1,2^. NHEJ ligates the DNA ends, but does so in an error-prone fashion^3^. Misrepair can lead to gross chromosomal translocations, amplifications of proto-oncogenes, or deletions of tumour suppressor genes, all of which can result in tumorigenesis and ultimately carcinogenesis^1,4^. HR uses the sister chromatid as a template to repair in an error-free fashion. Therefore, HR can only occur during the late S-/G2-phase of the cell cycle, when the DNA has been duplicated prior to cell division^3,5,6^. Two key players in the HR pathway are BRCA1 and BRCA2, which play a crucial role in recruiting the DNA recombinase RAD51 to the vicinity of the DSB. Localisation of RAD51 to the site of the DSB is essential for strand invasion of the sister chromatid and can be visualised through a RAD51 foci immunostaining. Knockdown of BRCA1 or BRCA2 leads to loss of these RAD51 foci^6,7^. Germline mutations in *BRCA1* or *BRCA2* are associated with a highly increased risk for breast and ovarian cancer^6^.

Zebrafish (*Danio Rerio)* contains orthologues for many genes involved in DNA repair pathways that are available in higher eukaryotes, including the *brca2* gene^8–10^. Despite its poor overall homology with human *BRCA2* (22%), the functional N-terminal transcription activation domain, C-terminal DNA binding domain and RAD51 binding BRCA repeats are conserved in the zebrafish Brca2 protein^9,10^. It was shown that zebrafish *brca2* acts as a caretaker gene and is involved in maintaining genomic integrity through HR^9,10^. Brca2 deficient zebrafish models were shown to be sensitive to mitomycin C (MMC), a DNA crosslinker for which repair through HR is essential, and develop an all-male phenotype due to failure of meiotic recombination during the sex determination period^9,10^. Human *BRCA2* ^−/−^ patients develop Fanconi Anaemia, which is characterised by genomic instability and sensitivity to MMC^11^. Liu *et al*. provided evidence that zebrafish Rad51 is essential for HR in zebrafish, as morpholino mediated knockdown of this protein resulted in decreased HR^12^.

So far, up to 20 Fanconi Anaemia (FA) genes have been identified (*FANCA*, *B*, *C*, *D1* (*BRCA2*), *D2*, *E*, *F*, *G* (*XRCC9*), *I*, *J* (*BRIP1*), *L*, *M*, *N* (*PALB2*), *O* (*RAD51C*), *P* (*SLX4*), *Q* (*ERRC4*), *R* (*RAD51*), *S* (*BRCA1*), *T* (*UBE2T*) and *U* (*XRCC2*))^13^. With the notable exception of *BRCA1*, zebrafish has the complete set of FA genes at its disposal^14^. The apparent absence of zebrafish *brca1*, a gene with a plethora of important functions, suggests that some modifications between the human and zebrafish HR repair pathway have occurred during evolution^14,15^.

Despite the importance of HR in maintaining genomic stability, not much is known about this pathway in zebrafish^8^. As zebrafish is an increasingly important model organism for studying cancer (pathway discovery, compound screening, drug discovery, …) it is crucial to unravel how this repair pathway functions in zebrafish^16,17^. Therefore, we developed the Rad51 foci assay in zebrafish, enabling the accurate measurements of *in vivo* HR activity under a wide range of experimental conditions.

## Results

### Determination of proliferative tissues in 72 hpf zebrafish embryos

**Figure 1:**
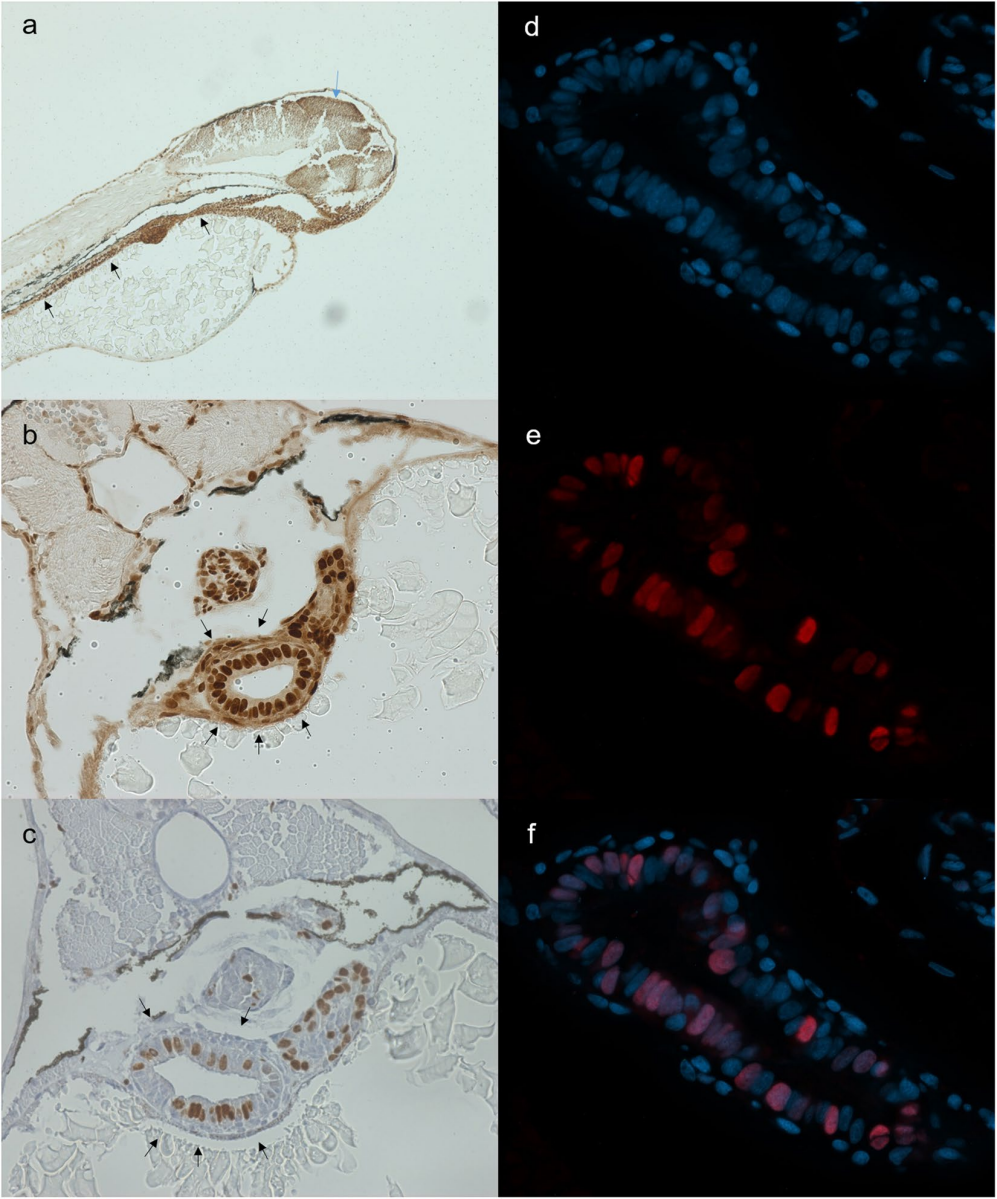
Stainings for PCNA (**a**,**b**), BrdU (**c**) and geminin (**d-f**). All stainings were performed on 72 hpf embryos. (**a**) Sagittal section stained for PCNA. Strong staining is found in the tectum (blue arrow) and gastro-intestinal tract (black arrows). (**b**) Transversal section stained for PCNA. PCNA stains positive in the gut tube, swim bladder and liver. (**c**) Transversal section stained for BrdU. BrdU intake is prominent in intestinal cells. (**d**–**f**) Separate images of nuclear staining (DAPI) (**d**), mCherry (zGem signal) (**e**) and overlay (**f**). (**f**) mCherry signal is present in a large proportion of intestinal cells.

As HR can only occur in dividing cells, it is essential to target a tissue that has high proliferative capabilities. Sagittal and transversal sections of 72 hours post fertilisation (hpf) wild type embryos were made and stained for Proliferating Cell Nuclear Antigen (PCNA), which stains cells in G1-, S-, G2- and M-phase of the cell cycle and acts as a general marker for dividing cells. PCNA is most noticeable in the tectum and throughout the intestinal tract (Fig. 1a,b). All nuclei in the intestinal tract show strong PCNA staining, which indicates the high proliferative capacity of these cells at 72 hpf. Due to the prominent staining and large morphology of the nuclei, we focused on the intestinal tract for further stainings.

To further confirm the proliferative capacity of the intestinal tract, embryos were administered Bromodeoxyuridine (BrdU) at 72 hpf for 2 hours and subsequently fixed and stained with an anti-BrdU antibody. BrdU is an analogue of thymine and will be incorporated into the DNA of cells during the S-phase. Transversal sections reveal that throughout the gastro-intestinal tract, cells incorporate BrdU (Fig. 1c). Quantification of the number of BrdU + cells show that up to 40% of the intestinal epithelial cells have incorporated BrdU, indicating that these cells are actively going through S-phase.

In a third experiment, we examined the intestinal tract of 72 hpf transgenic *Tg(EF1a: mCherry-zGem)oki011* zebrafish, which expresses an mCherry-fused 100-amino acid peptide of geminin under the control of the EF1α promotor and allows for fluorescent visualisation of the nuclei of cells that are in late S-/G2-phase of the cell cycle^18,19^. Since the mCherry signal is destroyed during the fixation process, an immunostaining with an anti-mCherry antibody was performed. Geminin positive cells were clearly visible in and around the intestinal tract (Fig. 1d–f). Quantification of intestinal cells in three embryos shows that on average 40–60% of the nuclei in the intestinal tract were positive for geminin.

The three different markers (PCNA; BrdU; geminin), therefore, manifest the proliferative nature of the intestinal tissue in zebrafish embryos. Moreover, a large proportion of the cells in the intestinal tract are in late S-/G2-phase of the cell cycle, which are the phases where HR is active. As such, this tissue was chosen for optimisation of the Rad51 foci assay.

### Wild type embryos show Rad51 foci upon irradiation

**Figure 2:**
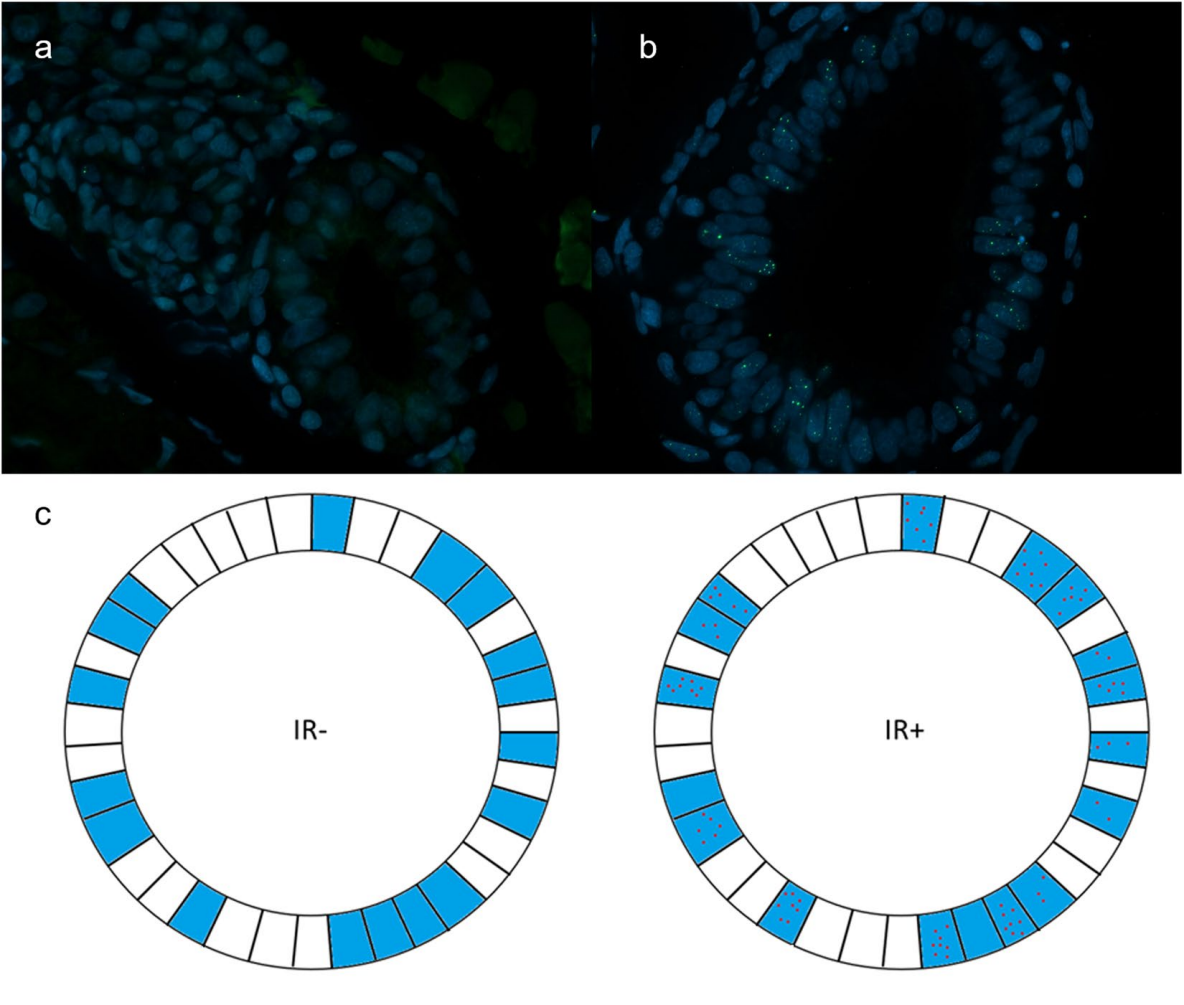
Representative images of Rad51 staining. Rad51 foci are absent in unirradiated sample (**a**), while irradiated sample displays clear Rad51 foci (**b**) (green fluorescence). (**c**) Schematic drawing of an unirradiated (left) and irradiated (right) gut tube section. Cells in blue are in late S-/G2-phase and thus capable of HR. Cells in white are not capable of HR and will never show Rad51 foci. Red dots indicate Rad51 foci. Quantifying all cells would lead to a serious underestimation of your signal, due to the large proportion of non-HR capable cells. Instead, quantifying only cells containing foci will give a more true value for irradiated conditions. To estimate the amount of HR capable cells in unirradiated samples, one can extrapolate the percentage of cells that contain Rad51 foci from the irradiated samples.

To optimise conditions for visualisation of Rad51 foci, a time kinetics experiment was performed. Embryos at 72 hpf were irradiated with 10 or 20 Gy and fixed at 1, 3, 5, 7 and 9 hours post irradiation (hpi). Unirradiated controls at 5 and 9 hours were also included. Transversal sections of the intestinal tract were made. To show that irradiation with 10 and 20 Gy induces DSB, immunostaining for γH2AX, a DSB marker, was conducted. An abundance of nuclear γH2AX foci was detected in irradiated embryos, but not in unirradiated controls (Supplementary Fig. S1). Staining for Rad51 showed formation of Rad51 foci in irradiated embryos while unirradiated controls contain almost no Rad51 foci (Fig. 2a,b).

Considering all intestinal cells to determine the mean number of foci/cell would result in an underestimation of Rad51 foci, as geminin staining showed that a substantial proportion (~40–60%) of these cells are not in late S-/G2-phase. Instead the following method was developed: as the embryos were irradiated with high doses, all cells have DSB (as observed with the γH2AX foci assay). Thus, we hypothesise that all cells that were in late S- or G2-phase at the moment of irradiation should show Rad51 foci and we restricted the quantification to cells containing foci.

1 $${\rm{Mean}}\,{\rm{foci}}\,{\rm{per}}\,{\rm{cell}}\,{\rm{in}}\,{\rm{irradiated}}\,{\rm{embryos}}=\frac{{\rm{Total}}\,{\rm{number}}\,{\rm{of}}\,{\rm{foci}}}{\#\mathrm{cells}\,{\rm{containing}}\,{\rm{foci}}}$$

This approach cannot be followed for unirradiated embryos (no DSB induced). Instead, we took the total number of foci and divided it by the expected number of cells that are capable of HR in these controls. This number was calculated by multiplying the percentage of Rad51 positive cells from irradiated conditions in the same experiment (typically 40–60%) with the total number of intestinal cells in the controls.

2 $${\rm{M}}{\rm{e}}{\rm{a}}{\rm{n}}\,{\rm{f}}{\rm{o}}{\rm{c}}{\rm{i}}\,{\rm{p}}{\rm{e}}{\rm{r}}\,{\rm{c}}{\rm{e}}{\rm{l}}{\rm{l}}\,{\rm{i}}{\rm{n}}\,{\rm{c}}{\rm{o}}{\rm{n}}{\rm{t}}{\rm{r}}{\rm{o}}{\rm{l}}{\rm{s}}=\,\frac{{\rm{T}}{\rm{o}}{\rm{t}}{\rm{a}}{\rm{l}}\,{\rm{n}}{\rm{u}}{\rm{m}}{\rm{b}}{\rm{e}}{\rm{r}}\,{\rm{o}}{\rm{f}}\,{\rm{f}}{\rm{o}}{\rm{c}}{\rm{i}}}{{\rm{ \% }}{\rm{e}}{\rm{x}}{\rm{p}}{\rm{e}}{\rm{c}}{\rm{t}}{\rm{e}}{\rm{d}}\,{\rm{H}}{\rm{R}}\,{\rm{c}}{\rm{a}}{\rm{p}}{\rm{a}}{\rm{b}}{\rm{l}}{\rm{e}}\,{\rm{c}}{\rm{e}}{\rm{l}}{\rm{l}}{\rm{s}}\,\ast \,{\rm{t}}{\rm{o}}{\rm{t}}{\rm{a}}{\rm{l}}\,{\rm{a}}{\rm{m}}{\rm{o}}{\rm{u}}{\rm{n}}{\rm{t}}\,{\rm{o}}{\rm{f}}\,{\rm{c}}{\rm{e}}{\rm{l}}{\rm{l}}{\rm{s}}}$$

**Figure 3:**
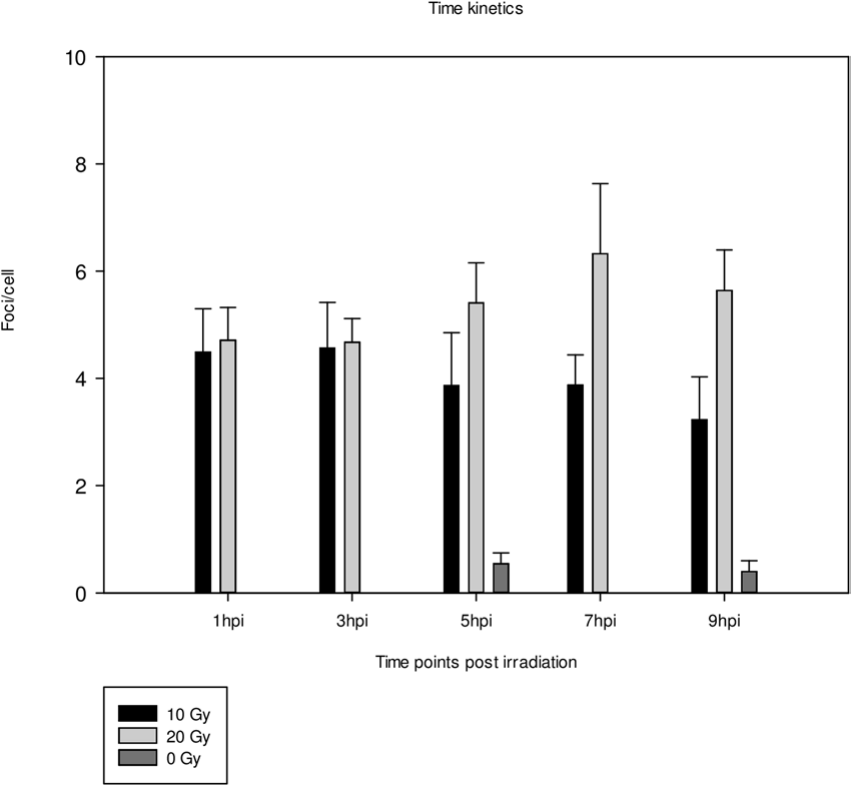
Time kinetics experiment. Graph displaying the number of foci/cell in function of different time points. Three embryos per condition were irradiated with 10 or 20 Gy and fixed at different time points, ranging from 1–9 hours. Unirradiated embryos were also fixed at 5 and 9 hours. Error bars display 95% CI.

For convenience purposes, the values obtained in irradiated and unirradiated embryos will always be referred to as ‘foci/cell’. A visual representation of this quantification approach can be found in Fig. 2c. All irradiated embryos display distinct nuclear foci in intestinal cells while controls are almost completely devoid of Rad51 foci (Fig. 3; Supplementary Table S5). Two-way ANOVA did not indicate a difference in foci/cell between the different time points. However, the foci observed at 1 hour were more subtle, hampering adequate quantification, compared to later time points. This indicates that 1 hpi, Rad51 is still being recruited to the site of the DSB. For all subsequent experiments, embryos of 72 hpf were irradiated with 20 Gy, fixated at 5 hpi and sectioned.

Of note, in cells of the tectum and the retina, other highly proliferative regions of the zebrafish embryos 72 hpf, Rad51 foci are also visible. However, visualisation and adequate quantification of the number of Rad51 foci in these regions is challenging due to the smaller size of the cells, the close proximity of the cells to one another and the difficulty for sectioning the correct region.

**Figure 4:**
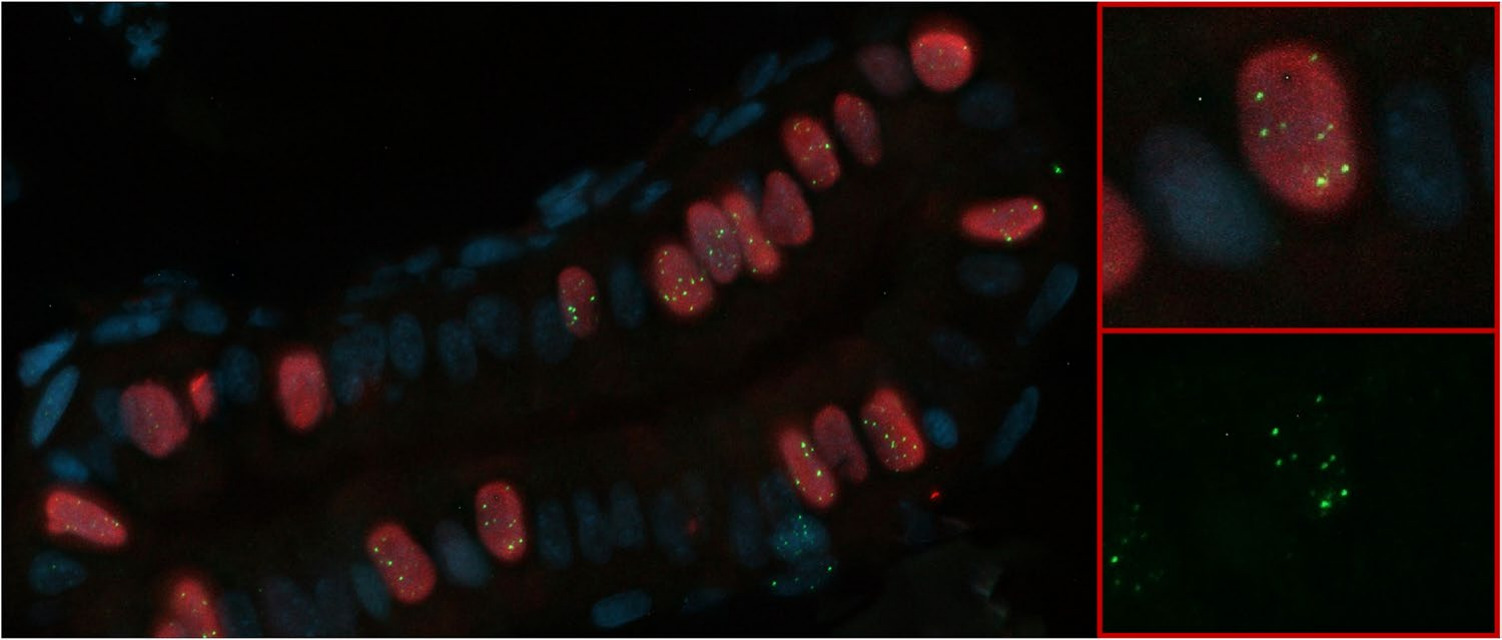
Rad51/geminin co-staining of irradiated embryo. Rad51 foci (green) are almost exclusively found in geminin positive nuclei (red).

The quantification method used in the time kinetics experiment was based on the assumption that all cells in late S-/G2-phase repair a number of DSB by means of HR and thus show Rad51 foci after irradiation. To confirm that this quantification is reliable, three transgenic *Tg* *(EF1a* *: mCherry-zGem)oki011* embryos were irradiated with 20 Gy, fixed at 5 hpi and stained for both Rad51 and mCherry (zGem) (Fig. 4). Unirradiated embryos were also included.

Quantification was conducted in two ways. First, all cells containing foci were included (disregarding if cells stained positive for geminin or not) and the mean number of foci/cell was calculated using formulas (1) and (2). In Supplementary Fig. S2 and Supplementary Table S6 these “hypothesised values” are shown. Second, only the geminin positive cells were included and the number of foci divided by the amount of geminin positive cells was calculated. These data are indicated in Supplementary Fig. S2 and Supplementary Table S6 as“true values”. For both the irradiated and unirradiated embryos, the “hypothesised value” correlates very well with the “true value” (Irradiated: 6.14 versus 6.06 foci/cell respectively; unirradiated: 0.67 vs 0.47 foci/cell respectively, Supplementary Table S6), confirming the validity of our quantification method.

### Knockout/knockdown of Brca2 results in absence of Rad51 foci

**Figure 5:**
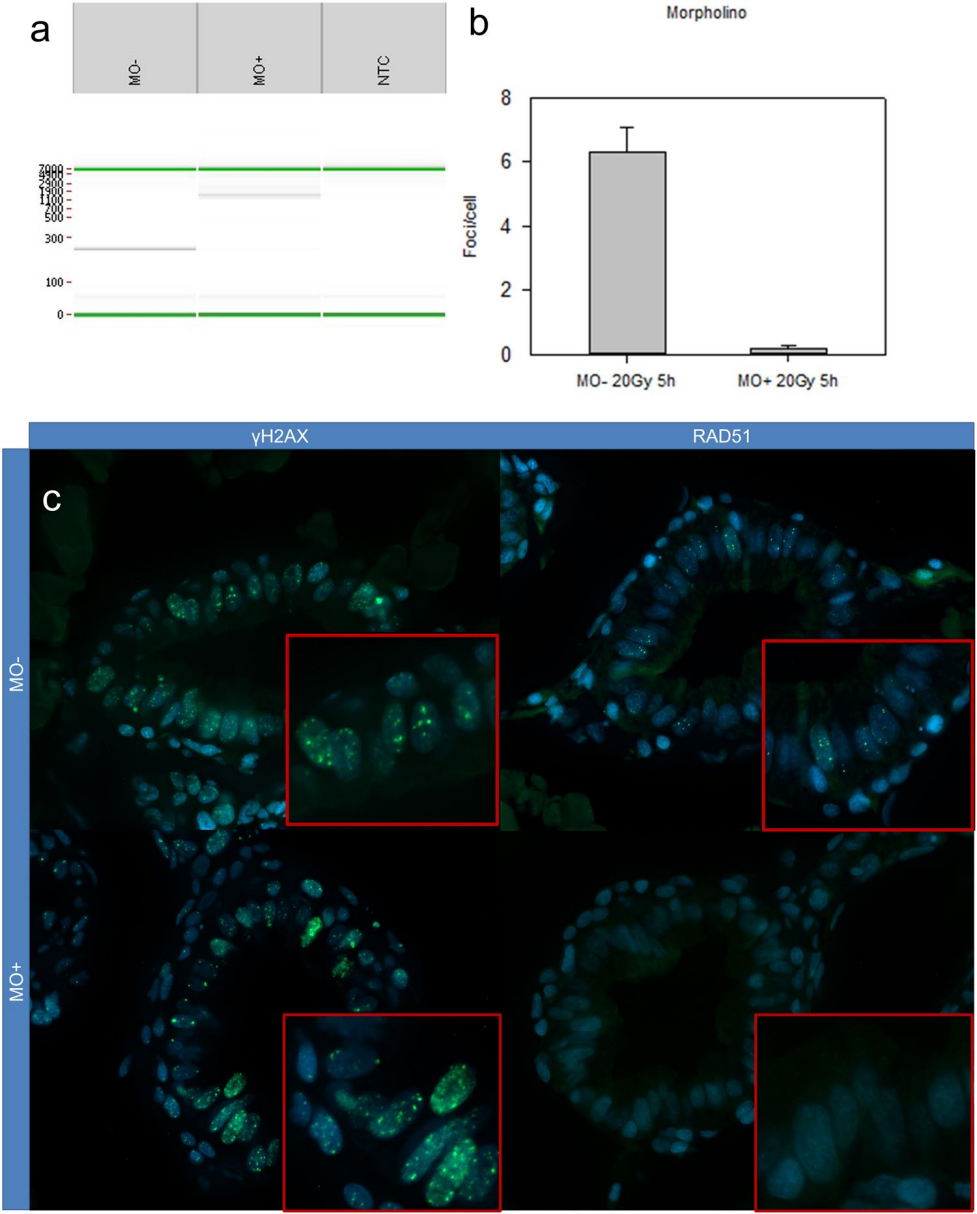
Results of morpholino experiment. (**a**) Digital gel of RT-PCR (obtained by Labchip®GX software) of uninjected (MO−) and injected (MO+) embryos. Injecting the morpholino results in retention of intron 7, resulting in a transcript containing a premature stop codon. This is visualised as a longer RT-PCR fragment, using primers located in exon 4 and 8 (expected size: 250 bp; in case of intron 7 retention: 1600 bp). In the morphants, there is a clear band of 1600bp, indicative for intron retention. (**b**) Quantification of Rad51 foci in three morphants (MO+) and uninjected (MO−) embryos. Almost no Rad51 foci are present in the morphants. Error bars display 95% CI. (**c**) γH2AX foci are present in both irradiated wild types (upper left) and morphants (lower left). Wild types display clear Rad51 foci (upper right), while morphants display no Rad51 foci.

A *brca2* zebrafish knockdown model was obtained by injecting 1-cell stage wild type embryos with a splice-blocking morpholino targeting the exon7/intron7 splice site of *brca2*. At 72 hpf, embryos were irradiated with 20 Gy, followed by fixation at 5 hpi. Three uninjected embryos (MO−) were also irradiated. RT-PCR was performed to check the functionality of the morpholino. Control embryos show normal amplification of a region between exon 4 and exon 8 (250 bp). Injection of the morpholino causes a band shift to about 1600 bp, corresponding to an intron 7 retention (Fig. 5a), and multiple stop codons are predicted within the first 50 bp by this intron retention.

In morphants, a severe loss of the number of Rad51 foci/cell was noticed when compared to controls (6.3 versus 0.2 foci/cell; p = 10^−4^) (Fig. 5b). To confirm that DSB were induced in both groups, the γH2AX foci assay was performed. Both wild types and morphants show an abundance of γH2AX foci compared to unirradiated embryos (Fig. 5c).

**Figure 6:**
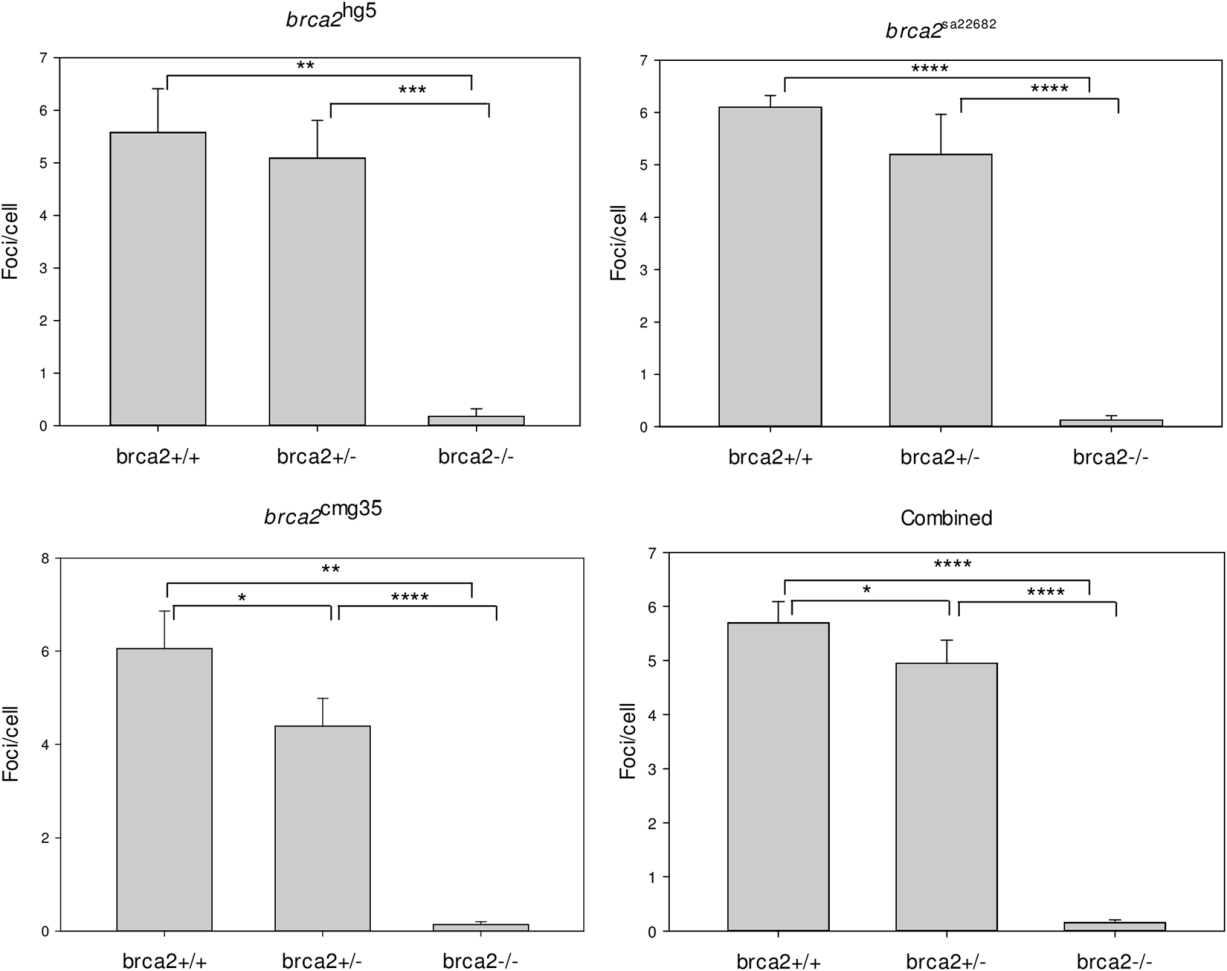
Rad51 foci quantification of *brca2* ^sa22682/+^, *brca2* ^hg5/+^ and *brca2* ^cmg35/+^ in-cross. At least three embryos per genotype were stained. All *brca2* ^−/−^ lines show a complete absence of Rad51 foci. *brca2* ^+/+^ embryos display more Rad51 foci than *brca2* ^+/−^. This difference is statistically significant in *brca2* ^cmg35^ or when combining data from all lines. *p < 0.05; **p < 0.01; ***p < 0.001; ****p < 0.0001.

In addition to the *brca2* MO-mediated knockdown model, three different knockout lines for *brca2* were tested with the Rad51 foci assay: *brca2* ^sa22682^, *brc2* ^hg5^ and *brca2* ^cmg35^ (in-house developed). As *brca2* ^−/−^ fish develop to infertile males, most likely due to apoptosis of oocytes during the critical period of sex determination of zebrafish, these homozygotes cannot be used for propagation^9,10^. This ‘all-male’ phenotype was confirmed in our in-house developed *brca2* ^cmg35^ line, where 16 adult *brca2* ^−/−^ fish developed exclusively as males (chi², p = 6.10^−5^). In addition, using offspring from *brca2* ^−/−^ fish is discouraged as these fish would have a dysfunctional HR pathway and may present with genomic instability. This predisposition could be transmitted to their offspring and affect results. Therefore, in this experiment, heterozygous carriers of each knock-out line were in-crossed and resulting embryos were irradiated with 20 Gy and fixed at 5 hpi. At least three embryos per condition were quantified. For all lines, Rad51 foci were present in both *brca2* ^*+/+*^ and *brca2* ^+/−^ embryos, whilst being absent in *brca2* ^*−/−*^ embryos (Fig. 6; Supplementary Table S7). Interestingly, a statistically significant difference was also observed between *brca2* ^*+/+*^ and *brca2* ^+/−^ in the *brca2* ^cmg35+/−^ line, with less foci being present in the *brca2* ^+/−^ condition (p = 0.02). The two other lines showed a similar trend but results were not significant. When combining the data from all three lines, the difference between *brca2* ^*+/+*^ and *brca2* ^+/−^ was statistically significant (p = 0.02).

## Discussion

Zebrafish is an increasingly popular model system in genetic and cancer research because of its relative small size, ease of housing, *ex utero* development and initial transparency. However, current knowledge on the HR repair pathway of this model organism is limited^8,20^.

In this work, we aimed to develop an assay to accurately quantify HR activity in zebrafish through means of Rad51 foci quantification. Furthermore, using this method, we wanted to determine if zebrafish Brca2 conserved its function in recruitment of Rad51 to the site of DSB.

To be able to study HR in zebrafish embryos, we first needed to identify a suitable tissue showing high HR activity. Through PCNA staining, we identified that intestinal cells 72 hpf are particularly highly proliferative. This confirms previous findings where it has been reported that between 26 hpf and 72 hpf the entire intestinal endoderm shows high proliferative capacity^21,22^. BrdU stainings performed on 72 hpf embryos indicated that about 40% of cells in the intestinal tract are in S-phase. In contrast, Wallace *et al*. found about 20% S-phase in intestinal cells in 72 hpf embryos. This might be attributed by differences between protocols, as they performed a 1 hour incubation while we applied 2 hours^23^. Finally, geminin stainings determined that about 40–60% of intestinal cells are in late S-/G2-phase at 72 hpf. The high number of cells in late S-/G2-phase makes intestinal tissue highly interesting for studies on HR because during these phases sufficient genetic material has been duplicated allowing the cell to repair DSB through HR^24^. Another advantage of intestinal tissue is the large size of the cells and their nuclei. This allows for adequate visualisation of discrete nuclei as well as Rad51 foci in these nuclei. Finally, the gut tube spans a large part of the embryo allowing the sectioning of intestinal cells to be straightforward. In conclusion, zebrafish embryonic intestinal tissue is an ideal target tissue when assessing HR functionality in this organism.

To activate the HR pathway, DSB were induced through ionising irradiation. A time kinetics experiment was performed, where 10 and 20 Gy were administered to 72 hpf embryos. A dose of 20 Gy and assessment of HR activity at 5 hpi was identified as optimal. Also, other studies reporting on radiation experiments in zebrafish, such as screening of radiation modifiers^25^ and engraftment experiments^26^, use up to 20 Gy. Studies where the RAD51 foci assay has been performed in human tumour biopsies and mammalian cell lines also use rather high doses (ranging from 5–12 Gy)^7,27,28^. Although a majority of the DSB are repaired through NHEJ, a sub-fraction of radiation-induced DSB are specifically repaired by HR. This is due to the complexity of the DSB repair resulting from increasing doses^29,30^. The Rad51 foci assay, therefore, requires high doses.

HR activity was quantified as the mean number of Rad51 foci/cell as described in the results section. This quantification was validated using the transgenic line *Tg(EF1a: mCherry-zGem)oki011*. Geminin is known to be active in the late S-/G2-phase and was previously shown to be an excellent HR marker in combination with RAD51 staining in human tumour tissues^7,31^. Of note, at any given dose and time point γH2AX foci are more prominent than Rad51 foci. This suggests that besides HR, other DSB repair pathways are also active. Liu *et al*. previously showed that NHEJ is the most dominant DSB repair pathway in zebrafish embryos^12^.

After optimizing the zebrafish Rad51 foci assay on wild type embryos, we determined if deficiency of Brca2 leads to absence of Rad51 foci recruitment to the DSB. Using a *brca2* MO-mediated knockdown, a complete loss of Rad51 foci was observed. However, as morpholinos may produce off-targeted effects^32^, we validated our results in three stable *brca2* mutant lines, harbouring different inactivating mutations in exon 8 (*brca2* ^cmg35^) or 11 (*brca2* ^sa22682^, *brca2* ^hg5^). For all stable lines, Rad51 foci were absent in mutants, while being present in both wild types and heterozygous carriers. We conclude that Brca2 has conserved its main function in recruiting Rad51 and that our assay can accurately quantify HR functionality. Morpholinos have the advantage that every injected embryo is deficient for Brca2, while only 25% of offspring from heterozygous carriers will be mutant. This makes the use of stable lines more labour intensive. However, morpholino-mediated knockdown is only temporary. The choice should be made in function of the data and experiments required.

Interestingly, for the *brca2* ^cmg35^ line we observed a borderline statistically significant difference in the number of Rad51 foci/cell between *brca2* ^+/+^ and *brca2* ^+/−^ embryos. This suggests that HR is less efficient in *brca2* ^+/−^ cells compared to *brca2* ^+/+^ cells. In general, tumours from patients with a germline *BRCA2* mutation contain an inactivating mutation of the second allele. However, in a considerable number of human *BRCA1/2* associated breast tumours, loss of heterozygosity (LOH) of the wild type allele is not observed^33–35^. It has been suggested that BRCA1/2 haploinsufficiency may cause genomic instability, initiating tumorigenesis^6,33,35^. Recently, a study on 160 *BRCA1* and *BRCA2* germline mutation-associated breast and ovarian cancer found absence of LOH in a subset of patients, in which they postulate haploinsufficiency as a possible mechanism for some of these cases^36^. Our data obtained in the zebrafish *brca2* ^+/−^ embryos indeed support reduced repair capacity through HR. The observed difference, however, is small (13% reduction). Nevertheless, a small reduction in HR could hypothetically lead to additional accumulation of mutations and eventually tumorigenesis in some cases. More studies are warranted to evaluate if the observed difference has an impact on repair capacity and how this can be translated to a tumorigenic process.

In conclusion, we have identified zebrafish embryonic intestinal cells as an ideal target to study the functionality of the HR pathway due to their highly proliferative activity and good morphology. Our study shows that zebrafish Brca2, like human BRCA2, plays a role in HR through recruitment of Rad51; furthermore, disabling Brca2 results in absence of Rad51 foci after irradiation. These results were observed in both morpholino injected embryos and in mutant zebrafish lines. We demonstrate here that zebrafish is a suitable model organism for studying HR functionality. Interestingly, the outcome of our experiments also suggests less efficient HR capacity in *brca2* ^+/−^ embryos. Further studies are warranted to evaluate if this leads to increased genomic instability due to haploinsufficiency.

We are convinced that, in the future, our Rad51 foci assay can be applied in combination with rescue experiments to study the pathogenic effect of *BRCA2* variants of unknown clinical significance^37^. Related to this, PARP inhibitors are a novel type of drug to selectively target HR deficient tumours through synthetic lethality^38^; our assay may be useful for testing the efficiency of new inhibitors. Another application may be related to evaluate the efficiency of Crispr-Cas9 mediated genome editing through HR in zebrafish.

## Material and Methods

### Zebrafish maintenance and lines

Zebrafish lines were housed in a Zebtec semi-closed recirculation housing system at a constant temperature (27–28 °C), pH (~7.5), conductivity (~550 µS) and light/day cycle (14/10). Fish were fed twice a day with dry food (Gemma Micro, Skretting) and once with artemia (Ocean Nutrition). The mutant *brca2* ^sa22682^ line was purchased from the European Zebrafish Resource Center (EZRC, Karlsruhe institute, Germany); *brca2* ^cmg35^ was generated using Crispr-Cas9 mutagenesis (cfr below); *brca2* ^hg5^ was a kind gift from Dr. Heather Shive (North Carolina State University)^9^; *Tg(EF1a: mCherry-zGem)oki011* was a kind gift from Dr. Ichiro Masai (Okinawa Institute of Science and Technology)^18^.

### Ethic statement

This study was approved by the local animal ethics committee (Ghent University hospital, Ghent, Belgium) application numbers: ECD 14/70 and 16/67 K. All methods were carried out in accordance with the approved guidelines.

### Crispr-Cas9 mutagenesis

Three sgRNA sequences targeting *brca2* were designed using CRISPRdirect^39^. From these sequences, synthetic dsDNA consisting of a 5′ random sequence, T7 promotor, target-specific sequence (minus PAM) and a constant region were designed according to Boel *et al*.^40^. These dsDNA sequences were ordered as G-blocks at Integrated DNA Technologies (IDT). G-blocks were dissolved in 20 µl nuclease-free water. PCR conditions can be found in Supplementary data (Supplementary Table 2–4).

PCR product was purified with the Qiaquick PCR purification kit (Qiagen) following general guidelines of the manufacturer. *In vitro* transcription was performed using the MEGAshortscript™ Kit (Thermo Fisher). The MEGAclear™ Kit (Thermo Fisher) was used to purify the transcription reaction. RNA quality was verified on the Experion (Bio-Rad). 25 pg sgRNA was co-injected with 250 pg Cas9 protein (Cas9 wild type nuclease protein with NLS, ToolGen) during the 1-cell stage. Phenol red 6% was used to visualise injection. At 24 hpf, 10 embryos were harvested for DNA extraction, using the KAPA Express Extract DNA Extraction kit. PCR was performed according to manufacturer’s protocol using primers flanking the crispr region. Samples were deep sequenced on the MiSeq and somatic indel frequency was analysed using BATCH-GE^40^. If a high out-of-frame indel percentage was identified, mosaic fish (F0) were grown to adulthood. These were out-crossed and offspring (F1) was tested for the presence of the mutation. If present, offspring was grown to adulthood and genotyped for the mutation.

### Morpholino injection

To knockdown zebrafish Brca2, a splice-blocking morpholino oligonucleotide (MO; Genetools) was designed targeting the exon7/intron7 boundary (5′-ATTGCgtatgtatgaatggtcttgc-3′) of *brca2*. At the 1-cell stage, wild type embryos were injected with 10 ng of the morpholino. The morpholino was dissolved in a 2% Phenol red solution for visualisation of injection.

### RNA extraction and RT-PCR

Ten embryos per condition were dechorionated and rinsed with PBS. 175 µl TRIZOL (Invitrogen) was added and embryos were homogenised. After 5 min incubation 70 µl of chloroform was added and samples were centrifuged for 15 minutes at 11000 rpm at 4 °C. The upper phase was transferred to a new tube and 100% ethanol was added. This mixture was brought onto a RNeasy mini-spin column (Qiagen) and centrifuged at 9600 rcf for 30 seconds. Samples were treated with 350 µl RW1 and briefly centrifuged. 35 µl RDD buffer and 5 µl DNaseI were added on each column and after 15 min incubation 350 µl RW1 was added, followed by a brief centrifugation. Samples were subsequently treated twice with 500 µl RPE buffer after which the RNA was finally eluted in 30 µl RNAse free water. cDNA synthesis was performed using the IScript™ cDNA Synthesis Kit (Bio-Rad). Samples underwent RT-PCR with primers surrounding the target area of the morpholino (F: 5′-CCTTGTTTGTTTGGCTCAGC-3′; R: 5′-CAGATGCAACCCGGTCC-3′). Sample sizing was conducted on the Labchip® GX capillary electrophoresis system.

### BrdU labelling

At 72 hpf, embryos were treated with 10 mM BrdU (Sigma-Aldrich) in E3 medium for 2 hours. BrdU was washed away prior to fixation.

### Irradiation and fixation

At 72 hpf, embryos were irradiated with the Small Animal Radiation Research Platform (SARRP, Infinity Lab, Ghent University). Embryos underwent irradiation with doses of 10 or 20 Gy X-rays (220 kV, dose rate 2.77 Gy.min^−1^). Embryos were euthanised and fixed for 1 hour in 4% PFA after different time points post irradiation (hpi) ranging between 1–9 hpi. Following fixation, embryos were placed in increasing alcohol concentrations and embedded in paraffin.

### Immunostainings

Paraffin embedded embryos were sectioned in 5 µm sections and placed on slides. The embryos were then deparaffinised and underwent 20 minutes of steam antigen retrieval in citric buffer (pH 6.1). For immunohistochemical stainings, slides were treated with 10% H_2_O_2_. Blocking serum was added to the samples to prevent aspecific binding. Slides were incubated with mouse anti-BrdU (1/100; Dako; M0744) or mouse anti-PCNA (1/100; Novo Castra; NCL-PCNA) for 2 hours. This was followed by incubation with biotinylated RAM (1/200, Dako) and Strep-HRP (1/200, Dako) antibodies for 30 minutes and 3,3′-diaminobenzidine tetrahydrochloride (DAB, Sigma-Aldrich) exposure for 10 minutes. Slides were counterstained with Mayer’s Haematoxylin (Merck).

For immunofluorescent stainings, slides were treated with anti-γH2AX (1/1000; Bethyl Laboratories; IHC-00059), anti-RAD51 (1/2000; Santa-Cruz, H-92) or anti-mCherry (Abcam; ab125096; 1C51) antibodies overnight at 4 °C. Slides were then incubated with Goat-anti-Rabbit Dylight 488 antibody (1/1000, Sigma-Aldrich) or Goat-anti-Mouse Dylight 594 (1/1000 Sigma-Aldrich). Finally slides were treated with DAPI + fluoromount (Sigma-Aldrich). For PCNA staining, no antigen retrieval or counterstaining was performed. Unless mentioned otherwise, three embryos per condition were used.

### Visualisation

Slides were scanned on a Zeiss Axio Observer.Z1 inverted microscope, using the Zen pro 2012 software. γH2AX, Rad51 and geminin images were captured using fluorescent microscopy. To allow for foci analysis (γH2AX, Rad51), a 100x enlargement was used and Z-stacks were made (0.22 µm thickness). Images were deconvoluted (fast iterative) and an orthogonal projection of 10 slices was performed. For the quantification of %BrdU positive cells, 236 intestinal cells were counted. For the quantification of %geminin positive cells, 1024 intestinal cells were counted. Quantification of Rad51 foci is explained in the results section.

### Genotyping

During sectioning of paraffin embedded embryos, some sections were reserved for genotyping. DNA was extracted with the Kapa Express Extract kit (Kapa Biosystems) and PCR according to the manufacturer’s protocol was performed. Inactivating mutations and primers used for genotyping *brca2* ^hg5^, *brca2* ^sa22682^ and *brca2* ^cmg35^ can be found in Supplementary Table S1. PCR products were Sanger sequenced to determine the genotype.

### Statistical analysis

Data was analysed using SPSS statistics 24 (IBM). Unless mentioned otherwise, groups were compared using the student’s t-test (two-sided, α = 0.05). Levene’s test for equality of variances was performed. Graphs were made with Sigmaplot (Systat Software Inc.).

### Data availability

All data generated or analysed during this study are included in this published article (and its Supplementary Information files).

## Electronic supplementary material

Supplementary information
