# Supplementary material for: Accurate quantification of homologous recombination in zebrafish: brca2 deficiency as a paradigm
Source: Sci Rep. 2017 Nov 28;7:16518. doi: 10.1038/s41598-017-16725-3 (PMC5705637; doi:10.1038/s41598-017-16725-3)

SUPPLEMENTARY INFORMATION

Accurate quantification of homologous recombination in zebrafish: brca2 deficiency as a paradigm

Jeroen Vierstraete[1,2], Andy Willaert[1,], Petra Vermassen[1], Paul J. Coucke[1], Anne Vral[2], Kathleen BM. Claes[1*]

[1]Center for Medical Genetics Ghent, Ghent University Hospital
[2]Department for Basic Medical Sciences, Ghent University

*Corresponding author
E-mail: Kathleen.Claes@UGent.be

Supplementary Table 1: Mutation and primers used for genotyping

Line	Mutation	Forward	Reverse
brca2^{hg5}	*c.1972C>T*<br>p.Q658X<br>exon 11	5’-CAAGTCAACGTCTATCACAACC-3’	5’-TGATGACTCTGAACTTCTCCC-3’
brca2^{sa22682}	*c.4006C>T*<br>p.Q1336X<br>exon 11	5’-TCTAGCTCCTCAGGGAAGCA-3’	5’-AGGTTCATGGTCCTTTGCAG-3’
brca2^{cmg35}	*c.534del13*<br>p.V178fs<br>exon 8	5’-TGTAGTAGGCTAAGCAAATACGG-‘3	5’-CAGAGTCCTTTGAGAGAGATGG-3’

Supplementary Table 2: PCR reaction mix

PCR component	volume/reaction (µl)
Kapa2G Robust MM (2x)	10
Forward primer (10µM)	0.6
Reverse primer (10µM)	0.6
H_2O	7.8
Dissolved G-block	1
Total volume/PCR reaction	20

Supplementary Table 3: PCR primers

Forward primer	5’-CCGCTAGCTAATACGACTCACTA-3’
Reverse primer	5’-AAAAGCACCGACTCGGTG-3’

Supplementary Table 4: PCR program

94° C	4 min	
94° C	30 sec	6 X (touchdown)
58° C	30 sec	
72° C	1 min	
94° C	40 sec	25 X
52° C	40 sec	
72° C	30 sec	
72° C	10 min	

Supplementary Table 5: Time kinetics experiment

	**Mean number of Rad51 foci/cell**		
	**10Gy (95% CI)**	**20Gy (95% CI)**	**0Gy (95% CI)**
**1h**	4.49 ± 0.81	4.71 ± 1.2	
**3h**	4.57 ± 0.85	4.68 ± 0.87	
**5h**	3.87 ± 0.99	5.41 ± 1.47	0.54 ± 0.21
**7h**	3.88 ± 0.56	6.33 ± 2.56	
**9h**	3.23 ± 0.8	5.64 ± 1.49	0.4 ± 0.2

Supplementary Table 6: Comparison of quantification

**Dose**	**Hypothesised value (95% CI)**	**True value (95% CI)**
20Gy	6.14 ± 1.36	6.06 ± 1.35
0Gy	0.67 ± 0.07	0.47 ± 0.04

Supplementary Table 7: Comparing the amount of Rad51 foci/cell between different genotypes for three mutations and combined

	**Mean number of Rad51 foci/cell**			
	brca2^{hg5}	***brca2***^{sa22682}	***brca2***^{cmg35}	**Combined**
brca2$^{+/+}$	5.58 ± 0.83	6.1 ± 0.22	6.05 ± 0.81	5.70 ± 0.39
brca2$^{+/-}$	5.09 ± 0.72	5.2 ± 0.76	4.39 ± 0.59	4.95 ± 0.43
brca2$^{-/-}$	0.18 ± 0.14	0.12 ± 0.08	0.13 ± 0.06	0.15 ± 0.05

Supplementary Fig S1: γH2AX staining. Unirradiated (upper) and irradiated (lower) gut section of 72 hpf embryo. Dose administered was 20 Gy, followed by fixation at 5 hpi.

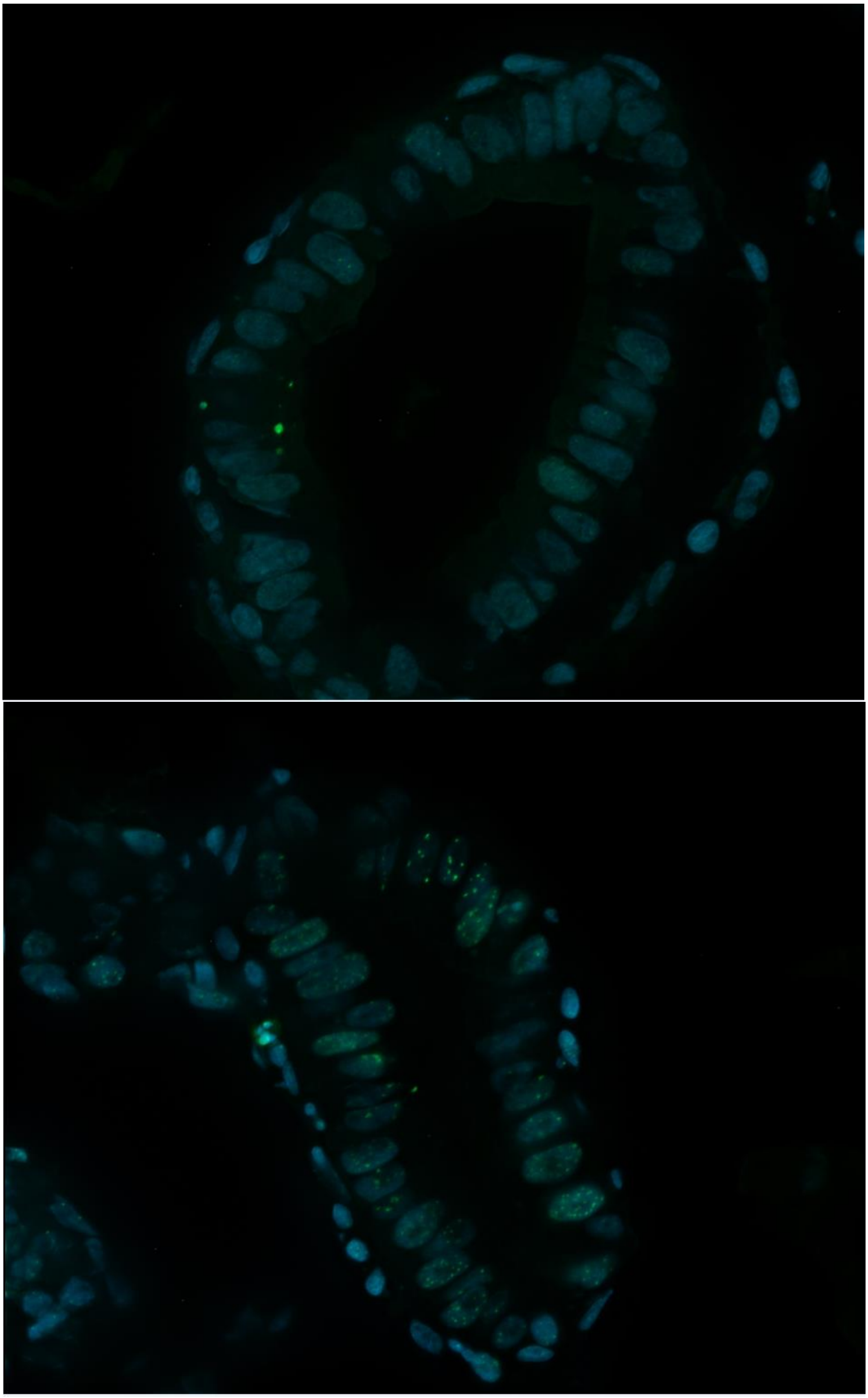

Supplementary Fig S5: Result of comparing quantification methods. Foci were either quantified according to the method used in the time kinetics experiment (hypothesised value) or by counting the mean number of foci in the geminin positive cells (true value). Values show Rad51 foci/cell ± 95% CI.

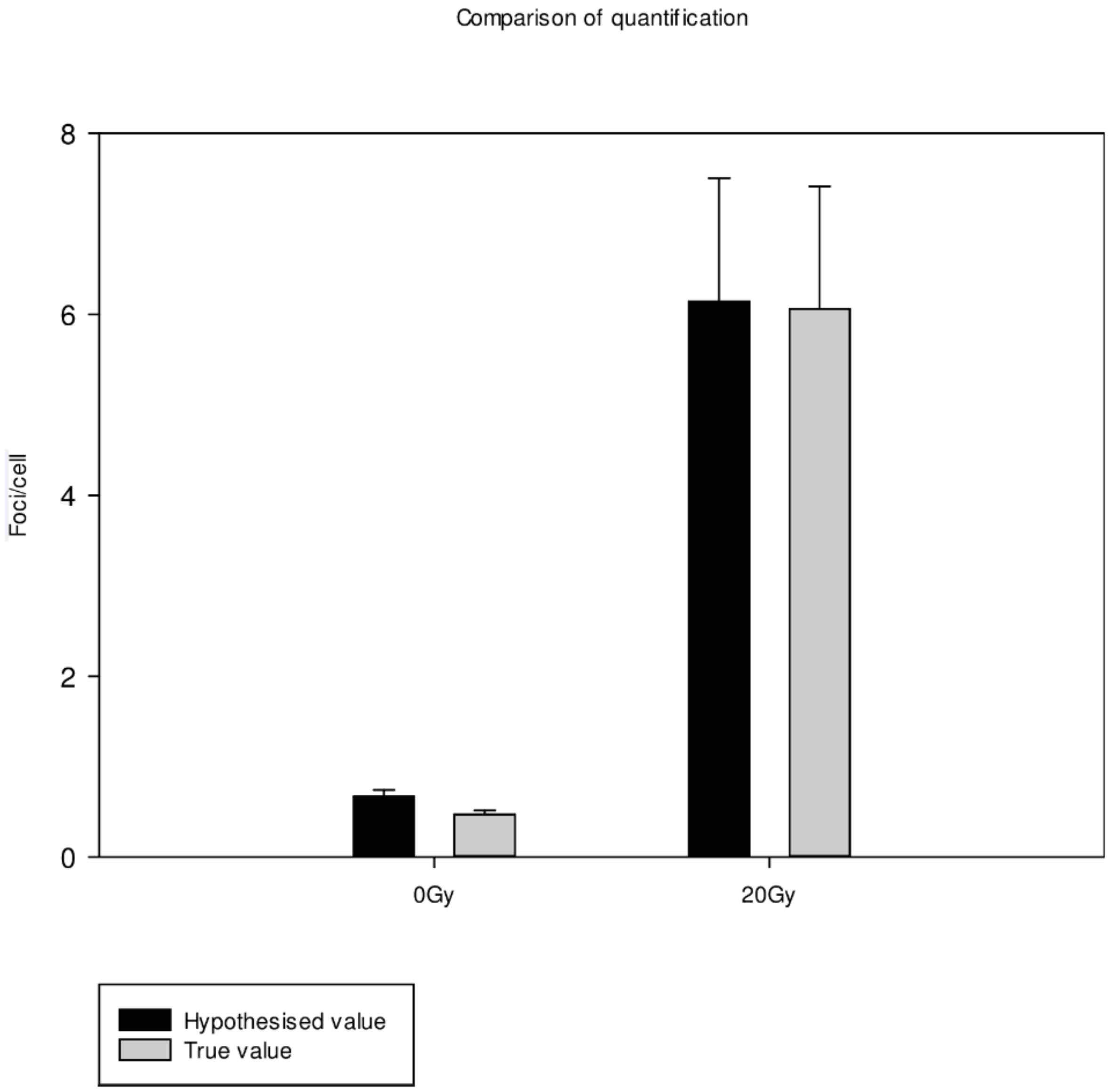

Supplement: Supplementary file 1 — Supplementary information [file 41598_2017_16725_MOESM1_ESM.pdf]
